# Mentoring the working nurse: a scoping review

**DOI:** 10.1186/s12960-020-00491-x

**Published:** 2020-07-29

**Authors:** Jerilyn Hoover, Adam D. Koon, Erica N. Rosser, Krishna D. Rao

**Affiliations:** 1 Credence Management Solutions, LLC, the Global Health Technical Professionals, USAID, 8609 Westwood Center Drive, Suite 300, Vienna, VA 22192 USA; 2 Department of International Health, Johns Hopkins Bloomberg School of Public Health, 615 N Wolfe St, Baltimore, MD 21205 USA

**Keywords:** Human Resources for Health, Mentoring, Nursing, Quality of Health Care, Rural Health

## Abstract

**Background:**

Mentoring programs for nurses already in the health workforce are growing in importance. Yet, the settings, goals, scale, and key features of these programs are not widely known.

**Objective:**

To identify and synthesize research on in-service nurse mentoring programs.

**Methods:**

We reviewed nurse mentoring research from six databases. Studies either referred explicitly to in-service nurse mentoring programs, were reviews of such programs, or concerned nurse training/education in which mentoring was an essential component.

**Results:**

We included 69 articles from 11 countries, published from 1995 to 2019. Most articles were from high-income countries (*n* = 46) and in rural areas (*n* = 22). Programs were developed to strengthen clinical care (particularly maternal and neonatal care), promote evidence-based practice, promote retention, support new graduate nurses, and develop nurse leaders. Of the articles with sufficient data, they typically described small programs implemented in one facility (*n* = 23), with up to ten mentors (*n* = 13), with less than 50 mentees (*n* = 25), meeting at least once a month (*n* = 27), and lasting at least a year (*n* = 24). While over half of the studies (*n* = 36) described programs focused almost exclusively on clinical skills acquisition, many (*n* = 33) specified non-clinical professional development activities. Reflective practice featured to a varying extent in many articles (*n* = 29). Very few (*n* = 6) explicitly identified the theoretical basis of their programs.

**Conclusions:**

Although the literature about in-service nurse mentoring comes mostly from small programs in high-income countries, the largest nurse mentoring programs in the world are in low- and middle-income countries. Much can be learned from studying these programs in greater detail. Future research should analyze key features of programs to make models of mentoring more transparent and translatable. If carefully designed and flexibly implemented, in-service nurse mentoring represents an exciting avenue for enhancing the role of nurses and midwives in people-centered health system strengthening.

The contents in this article are those of the authors and do not necessarily reflect the view of the U.S. President’s Emergency Plan for AIDS Relief, the U.S. Agency for International Development or the U.S. Government.

## Background

Several approaches to strengthening heath worker performance have been tried in a variety of settings [1]. Programs focused on financial incentives such as results-based financing or pay-for-performance have had limited success in sustaining provider behavior change [2]. Traditional training programs often place health workers in controlled environments during short trainings, which are not very effective for building lasting skills [3]. Centralized trainings in urban areas may particularly limit opportunities for rural health workers [4]. External trainings likely disrupt rural facilities, which may already struggle to provide regular access to care. There is mixed evidence that supportive supervision interventions, particularly in low-income countries, are effective [5]. Additionally, a recent systematic review of strategies to improve the practices of health care providers in low- and middle-income countries found combined training and supervision’s effect was larger than either intervention alone, but the quality of evidence was low to moderate [6]. Using job aids such as checklists to strengthen service delivery have also generated mixed results [7].

These challenges are attributable in part to clinical knowledge being difficult to translate into practice [8]. While health workers with more knowledge tend to provide better care, there is usually a gap between their knowledge and the care they provide [9]. Health worker motivation, support from colleagues, and work environment can impact this “know-do” gap [9]. Thus, traditional in-service training programs focused on didactic teaching are likely insufficient for strengthening the quality of clinical skills.

Systemic improvements to quality of care should include nurses because they shape primary care in profound ways [10]. Nurses and midwives make up nearly 50% of the world’s health workforce, promoting health, preventing disease, and delivering care in a variety of settings [11]. Nurses are essential to overall patient quality of care [12] and nurse staffing and education levels impact clinical outcomes [13, 14]. Yet, structural constraints, such as unsupportive work environments, frequently contribute to job-related stress, burnout, and turnover among nurses, with damaging effects on quality of care [15]. Therefore, practical strategies for effectively educating, supporting, and retaining nurses are prerequisites for universal health coverage [16].

Mentoring nurses already in the health workforce (in-service) is one such strategy. Although mentoring emerged in the nursing literature in the 1980s, there has been significant disagreement about the role of nurse mentors [17]. An interactive social process, mentoring is more expansive than didactic training, coaching, or supervision [4]. For this paper, we suggest that mentoring involves establishing “a relationship between two people that has the specific purpose of one assisting the other to grow and develop and to increase their role effectiveness.” [18]

Despite a lack of clear models for nurse mentoring and differing views about the degree to which the concepts of precepting, mentoring, and clinical supervision overlap [19], programs have been designed and implemented in low-, middle-, and high-income countries. If health officials are to consider in-service nurse mentoring as a viable workforce development strategy, they need a clear empirical basis for making decisions. There are no recent reviews about nurse mentoring specifically, and existing reviews are either out-dated [17] or define mentoring very narrowly [4]. We address this gap by identifying and synthesizing the literature about nurse mentoring in health service delivery settings. As researchers evaluating a large nurse mentoring program in a rural state in India, we were particularly interested in which countries and geographic settings were most represented in existing literature. We adopted a flexible and broad research question: “What is known from the existing literature about in-service nurse mentoring programs?” This analysis will shed light on the design and mechanics of nurse mentoring programs, including their aims, size, setting, and content.

## Methods

A literature review was conducted using Arksey and O’Malley’s methodology for scoping reviews [20]. While scoping reviews differ from systematic reviews, the Preferred Reporting Items for Systematic Reviews and Meta-Analyses (PRISMA) guidelines were consulted and adhered to (where applicable) to ensure rigor [21]. A research librarian was consulted, and six databases were searched in September 2019 using the terms: “nurs*” and “mentor*”. The terms “health care deliver*” or “quality improvement” were added to narrow results to those focused on practice settings (in-service) rather than pre-service nursing education settings. This produced 3491 articles, of which 2546 unique studies were included for screening. No language or date restrictions were applied, and results were limited to studies with abstracts. Controlled vocabulary terms for each database were used and the terms for “nurs*” and “mentor*” were emphasized when possible. See Table 1 for further details.

**Table 1:** Search strategy

Database	Search terms	Results	Without duplicates
PubMed	(Mentors[mesh] OR mentoring[mesh] OR mentor*[tw]) AND (Nurses[mesh] OR nurs*[tw]) AND (Delivery of Health Care[mesh] OR Healthcare Deliver*[tw] OR Health care deliver*[tw] OR Quality Improvement[mesh] OR Quality Improvement*[tw]))	1499	
EMBASE	('mentoring'/exp OR 'mentoring' OR 'mentor'/exp OR 'mentor' OR 'mentor*':ab,ti,kw) AND ('nurse'/exp OR 'nurse' OR 'nurs*':ab,ti,kw) AND ('health care delivery'/exp OR 'health care delivery' OR 'total quality management'/exp OR 'total quality management' OR 'quality improvement*':ab,ti,kw OR 'health care deliver*':ab,ti,kw OR 'healthcare deliver*':ab,ti,kw) AND [abstracts]/lim	984	631
CINAHL Plus	((MM "Nurses+") OR nurs*) AND ((MM "Mentorship") OR mentor*) AND ((MH "Health Care Delivery+") OR healthcare deliver* OR health care deliver*) OR ((MH "Quality Improvement+") OR quality improvement*))	513	326
Web of Science	TS = (mentor*) AND TS = (nurs*) AND TS = (delivery of health care* OR health care deliver* OR healthcare deliver* OR quality improvement*)	111	13
Scopus	(TITLE-ABS-KEY (mentor*) AND TITLE-ABS-KEY (nurs*) AND TITLE-ABS-KEY (("delivery of health care" OR "healthcare deliver*" OR "health care deliver*" OR "quality improvement*")))	277	30
PsychInfo	((MM "Mentor") OR mentor*) AND ((MM "Nurses" OR MM "Psychiatric Nurses" OR MM "Public Health Service Nurses" OR MM "School Nurses") OR (MM "Nursing") OR nurs*) AND ((DE "Health Care Delivery") OR (DE "Quality of Care") OR delivery of health care* OR healthcare deliver* OR health care deliver* OR quality improvement*)	107	47
**Total**			**2546**

**Fig. 1:**
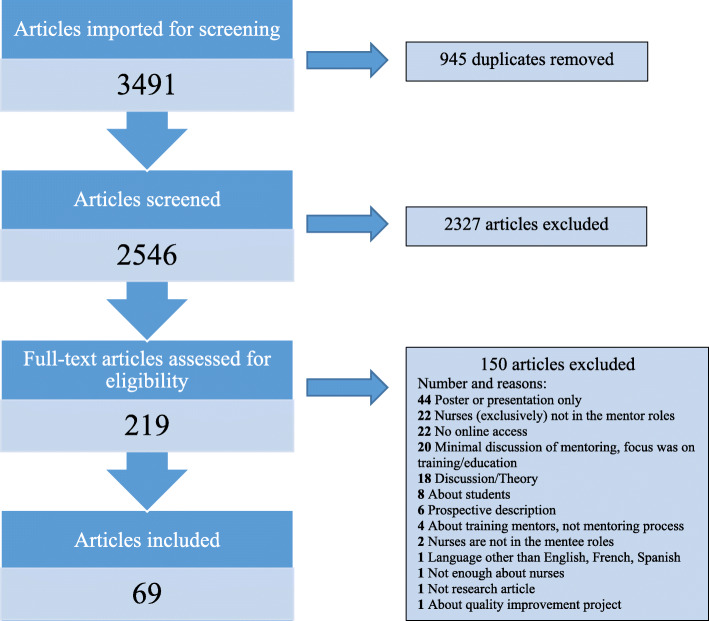
Flow diagram. This represents each stage in the literature review process, including screening articles by title, abstract, and full text. Reasons for exclusion during full-text review are also included

Covidence online software was used to organize, screen, and review all articles [22]. See Fig. 1 for review flow diagram. The 2546 articles were screened first by title and abstract; articles were required to have abstracts and mention a variation of “nurse” and “mentor” in the title or abstract. We included abstracts that referred to mentoring as a focus or essential part of their intervention. Included abstracts either referred explicitly to in-service nurse mentoring programs, were reviews of such programs, or were about nurse training or education interventions where mentoring formed a key part. Articles were excluded if nurses were not in mentor and mentee roles, or if they were about mentoring nursing students or faculty. Articles were excluded if they only referenced mentoring as a future recommendation. Articles were excluded if they were solely prospective or purely theoretical discussions of mentoring that did not reference implemented nurse mentoring. Narrative discussions about a single person’s experience were excluded. Studies were excluded if they were about mentoring a mix of professions where nurses were the minority, mentoring a multidisciplinary team, or mentoring a project. Based on these criteria, 2327 articles were removed.

We reviewed the full text of 219 articles and applied the exclusion criteria from the first round of screening. Articles were excluded based on earlier criteria if the mentors were not exclusively nurses (*n* = 22) or if the focus was on training or education with minimal discussion of mentoring (*n* = 20). Likewise, articles were excluded if they referred to a poster or presentation session only (*n* = 44) or lacked online access (*n* = 22). Four articles about training mentors, and not about the mentoring process, were removed. One article was excluded because it was not in English, French, or Spanish. Any conflicts were resolved by authors’ consensus. Using these criteria, 150 articles were excluded and 69 were included in the study.

Following the Arksey and O’Malley framework, we developed a table in Microsoft Excel to chart data about each article [20]. We included the article details, goal of the program, design features, program setting and size, and the format, frequency, and duration of mentoring contact. We also noted theoretical bases, the use of reflective practice, and the presence or absence of non-clinical mentoring. It was challenging to classify articles based on program geography. Using population estimates is difficult when comparing across countries where populations vary widely [23]. Given the limited information about program settings in many articles, we classified them into rural, urban, suburban, or a mix of settings based primarily on how authors identified the settings.

Consistent with the scoping review framework, this study does not attempt to make claims about the quality or weight of evidence. It is an overview of what is known about the research question based on each article. There were necessarily some subjective decisions made by the primary author about which themes to highlight. This was done in a reflexive manner in discussion among the authors. The authors of this paper represent diverse experience in nursing practice, public health practice and research, and policy research. This helped us develop a well-rounded understanding of in-service nurse mentoring literature.

## Results

Of the 69 included articles, 65% (*n* = 45) were published since 2010. Only 3% (*n* = 2) were published prior to 2000, 10% (*n* = 7) between 2000 and 2005, and 22% (*n* = 15) between 2006 and 2010. Articles were from 44 journals, 26 of which were nursing journals (determined by whether nursing was in the title). Sixteen of the included articles were about one of four mentoring programs; however, the articles focused on different iterations or facets of the interventions and mentoring was not always conducted in the same geographic areas. Because each article did not necessarily represent a distinct mentoring program or activity, results are reported on a per article, rather than a per program, basis.

**Fig. 2:**
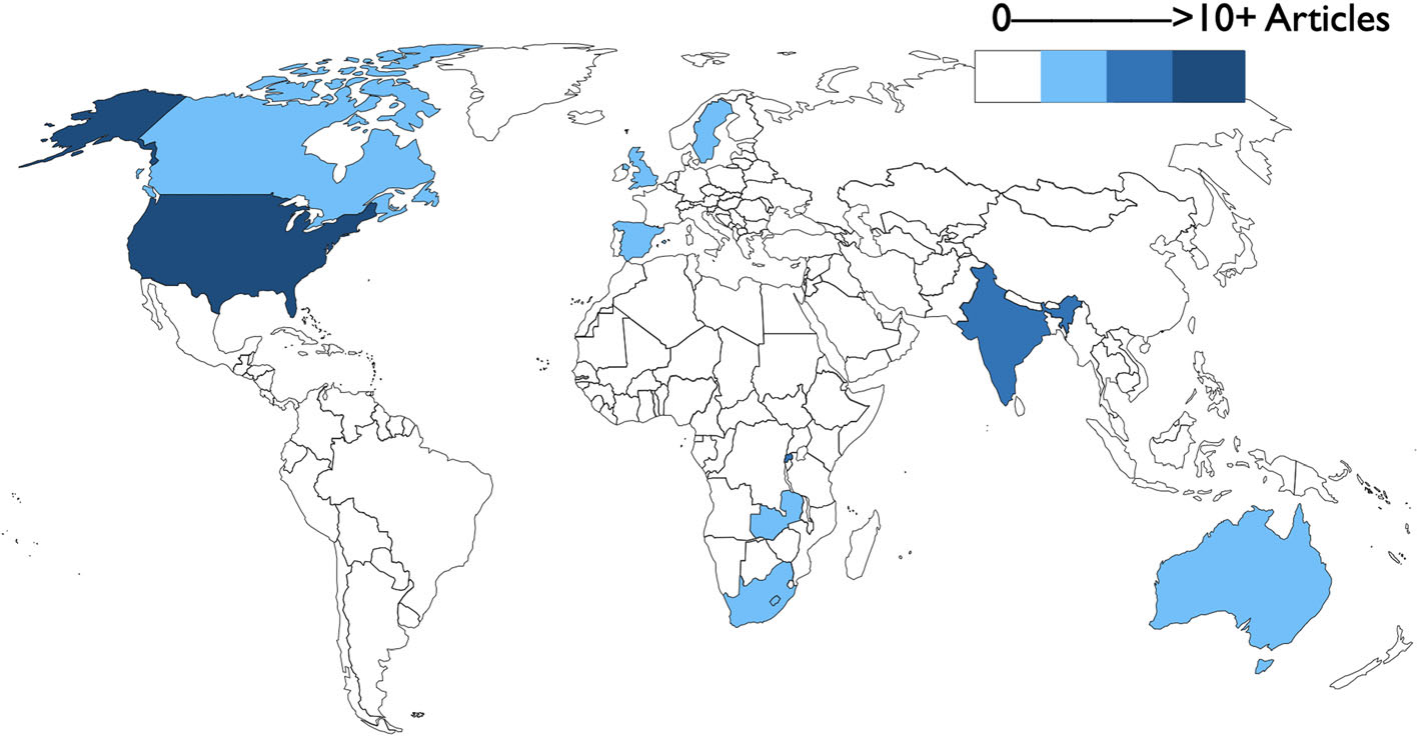
Literature heat map. This map illustrates the geographic location (country) in which the study took place. Lighter colors represent fewer numbers of articles and darker colors represent greater numbers of articles

Articles were categorized by country based on author locations and location of program implementation, which were the same for most articles. See Fig. 2 for an overview of the literature by country. For any articles where this was different, studies were categorized based on the country in which the nurse mentoring program took place. Research was conducted in 11 low-income, middle-income, or high-income countries (i.e., Australia, Canada, India, Lesotho, Rwanda, South Africa, Spain, Sweden, United Kingdom, United States of America, and Zambia). In total, 67% (*n* = 46) of the articles concerned programs in high-income countries. Nearly half of the studies (*n* = 33) were conducted in the United States. The next closest high-income country contributors were Australia (*n* = 5) and Canada (*n* = 4). Outside of the high-income countries, the countries contributing the most research were Rwanda (*n* = 9), India (*n* = 8), and South Africa (*n* = 4).

### Goals of the programs

Structured categories for goals of the nurse mentoring programs were developed from themes that emerged during the screening and review process (Table 2). Articles were sorted based on the primary purpose described for nurse mentoring, the most frequent of which was to strengthen a type of clinical care (*n* = 30). Among those articles, the most common types of clinical care targeted for improvement were maternal (including antenatal) and neonatal care (*n* = 10) and care for people living with the human immunodeficiency virus (HIV) (*n* = 7). The next most common goals were to promote evidence-based practice (*n* = 11), promote retention (*n* = 8), support new graduate nurse transition to practice (*n* = 8), and develop nurse leadership (*n* = 5).

**Table 2:** Overview of results

Overview of results	Number of articles (*N*=), percent of total (%)
**Goal of mentoring program**	***N*= 69**
Strengthen a type of clinical care	*N* = 30 (43%)
Promote evidence-based practice	*N* = 11 (16%)
Promote retention	*N* = 8 (12%)
New graduate nurse transition to practice	*N* = 8 (12%)
Leadership development	*N* = 5 (7%)
Other	*N* = 7 (10%)
**Scale of facility-based mentoring**	*N*= 55* *Articles about facility-based mentoring
One facility	*N* = 23 (42%)
2–10 facilities	*N* = 11 (20%)
10–50 facilities	*N* = 12 (22%)
50–100 facilities	*N* = 4 (7%)
Over 100 facilities	*N* = 5 (9%)
**Number of mentors**	***N*= 69**
0 to 10	*N* = 13 (19%)
10 to 50	*N* = 8 (11%)
50 to 100	*N* = 4 (6%)
Over 100	*N* = 4 (6%)
Unspecified/unable to determine	*N* = 40 (58%)
**Number of mentees**	***N*= 69**
0 to 10	*N* = 8 (11%)
10 to 50	*N* = 17 (25%)
50 to 100	*N* = 4 (6%)
Over 100	*N* = 9 (13%)
Unspecified/unable to determine	*N* = 31 (45%)
**Frequency of contact**	***N*= 69**
Unspecified	*N* = 27 (39%)
At least once (some multiple) within 1 month	*N =* 27 (39%)
At least once (some multiple) within 2 months	*N =* 10 (15%)
Reported based on total hours or contact time	*N =* 4 (6%)
Other	*N =* 1 (1%)
**Duration of mentoring**	***N =*69**
Under 3 months	*N =* 6 (9%)
3 months to 6 months	*N =* 7 (10%)
6 months to 1 year	*N =* 13 (19%)
1 year or longer	*N =* 24 (35%)
Unspecified	*N =* 19 (28%)

### Setting, scale, and design of the programs

Mentoring programs took place across a variety of settings and geographic areas. Articles were about programs in rural (*n* = 23), urban (*n* = 16), suburban (*n* = 2), or a mix of settings (*n* = 11); with many articles (*n* = 17) not providing this information. Most programs were in health care facilities and the majority of articles (*n* = 55) specified the number of health care facilities in which mentoring occurred. Others took place within a health system and did not specify the number of facilities (*n* = 2). Additional articles were about public health or community settings (*n* = 2) or were based primarily on the location of the mentees (*n* = 2). For the remaining articles (*n* = 8), the setting was unspecified or could not be determined based on the article. Nearly half of the articles (*n* = 30) were about programs developed internally to the setting where mentoring took place. The majority (*n* = 39) were about programs developed with external support, such as partnerships with academic institutions, nursing associations, and/or nonprofit organizations.

### Who were the mentors and mentees?

Most of the articles described nurses in mentor roles as “experienced,” “senior,” “expert,” or “graduate educated.” A few studies (*n* = 5) either specifically chose mentors who were not in supervisory roles over mentees or included feedback from mentees who felt they could discuss more openly with non-supervisory mentors. But nearly as many articles (*n* = 4) used “nurse managers” or “charge nurses” as both supervisors and mentors.

Most of the papers (*n* = 42) described mentees as “staff nurses,” “clinical nurses,” “facility nurses,” or similar. Additional articles (*n* = 17) reported programs specifically focused on newly graduated nurses new to clinical practice, new hires, or those re-entering the workforce. Several others (*n* = 10) described mentees as experienced nurses, leaders in practice settings, or advanced practice nurses. The programs focused on new graduates, new hires, or returning to work nurses were exclusively in high-income countries—the United States (*n* = 15), Canada (*n* = 1), and Sweden (*n* = 1). Categorizing levels of nurse mentor and mentee education or experience was difficult because of the diversity among the program contexts. For example, a Bachelor of Science (BSc) is common entry-level education for nurses in many high-income countries while in low- and middle-income countries, often only managers or higher-level nurses have BSc education.

### How many mentors and mentees?

Many of the 69 included articles did not specify the number of mentors (*n* = 40) or the number of mentees (*n* = 31) or these numbers could not be determined. In general, the numbers of mentors and mentees reported by the studies were fairly small, with 10 or fewer mentors and 50 or fewer mentees. Of the 29 articles which reported the number of mentors, 45% (*n* = 13) were about programs with 10 or fewer mentors. Of the 38 articles which reported the total number of mentees, 66% (*n* = 25) were about programs with 50 or fewer mentees. In some cases, articles reported a range (e.g., one to three mentees per health care facility) rather than a total number. For these articles, the range of possible mentees was calculated based on what the authors reported and the number of health care facilities. The median of this range was used to determine into which mentee size category to place the article.

### Frequency of contact

Many studies did not specify the frequency of contact between mentors and mentees. It was either not addressed or reported as left to mentors and mentees to decide. For articles reporting on programs specifying frequency of contact (*n* = 38), the most common was at least once per month (*n* = 27) followed by at least once within 2 months (*n* = 10). A few studies (*n* = 4) reported only the total hours of mentoring time per mentee.

### Duration of mentoring

The mentoring activities described varied greatly in duration. Many of the included studies (*n* = 19) did not specify the duration of mentoring, or it could not be determined from the article. Of studies which did report on the duration of mentoring (*n* = 50), the length of time reported was less than 6 months (*n* = 13), between 6 months and 1 year (*n* = 13), or 1 year or longer (*n* = 24).

### Type of contact

The high degree of variation in how the included studies described mentoring and the lack of clear description in many articles made it impossible to classify the studies into categories based on types of mentoring contact. Articles reported on in-person mentoring as well as distance mentoring or e-mentoring. Articles referenced individual mentoring, group mentoring, and a mix of both.

Fewer than half of the studies (*n =* 29) discussed the use of reflective practice as part of their programs. Among the programs using reflective practice, the most common description was the review of real cases or existing processes in the health facilities. Nurse mentees engaged in critical reflection to identify potentially flawed decision making and opportunities to improve care. Some articles (*n =* 5) discussed how mentees validated their decisions through discussions with their mentors or their peers.

Most of the studies (*n =* 36) described mentors providing only clinically focused mentoring. The articles which describe mentoring activities outside of a clinical focus (*n =* 33) mentioned social or other professional emphases. Social activities included mentors providing emotional support or socializing mentees into the working environment to supplement clinical learning [24]. One study emphasized the trust built through relationships between mentors and mentees as the foundation for mentee learning [25]. Professional activities included career advice and support, networking, and supporting nurse mentees to interpret and use research in their practice or create new research to build evidence-based practice.

### Theory

We recorded which programs reported an explicit theoretical basis. Only six articles reported their mentoring programs to be based on established social theory. Of those, two referenced Benner’s theory of novice to expert [26], two referenced Roger’s theory of diffusion of innovations [27], one referenced Bandura’s theory of self-efficacy [28], and one Gustafsson’s Sympathy-Acceptance-Understanding-Competence model for confirming mentorship [29, 30]. While not a theoretical basis, an additional six articles referenced the World Health Organization’s clinical mentoring guidelines for task-shifting for HIV prevention and treatment [31]; this included programs which were not about mentoring for HIV care delivery.

## Discussion

The fact that 65% (*n =* 45) of the articles included in this scoping review were published since 2010 demonstrates this is an emerging field of inquiry and programmatic experimentation. The geographic spread of the literature shows that interest emanates from high-income countries, and particularly the United States. This may be driven in part by more established traditions of nursing scholarship and advanced nursing education. However, this trend appears to be changing quickly. Rwanda, India, and South Africa contributed 30% (*n =* 29) of the included articles. Of those, 15 were published from 2015 to 2019. In Rwanda and India, this literature is based on large-scale nurse mentoring programs in rural settings implemented through partnerships between national or state governments and nonprofit organizations. There were multiple articles about different iterations of these programs or facets of the mentoring interventions. In Rwanda, the *Mentoring and Enhanced Supervision at Health Centers (MESH)* program was implemented by Partners in Health in partnership with the Rwandan Ministry of Health [25, 32–36]. In India, programs were in Bihar and Karnataka states. CARE India has partnered with the Government of Bihar to implement a mobile nurse mentoring program and the *AMANAT* program whose various iterations have provided clinical mentoring for nurse midwives in more than 320 primary health centers [3, 37–40]. In Karnataka, the Karnataka Health Promotion Trust and international partners mentored nurses in up to 385 primary health centers to improve the quality of maternal and neonatal care [41–43]. Aside from the programs in Rwanda and India which were featured in multiple articles, two articles were about a nurse mentoring program to promote retention at the Banner Good Samaritan Medical Center in Phoenix, Arizona [44, 45].

Of articles which included information about the setting of the nurse mentoring programs, the most common locations were rural areas. In addition, the three large programs in Rwanda and India were all implemented in areas authors identified as rural. This suggests that practitioners view nurse mentoring as a suitable intervention to improve clinical knowledge and ability of rural nurses. This may be because rural nurses might have less access to training and continuing education than their urban counterparts.

The most frequently cited program goal across all articles was to strengthen clinical care, particularly antenatal, maternal, and neonatal care, and HIV care; emphases which may reflect either need and/or higher availability of resources for these types of care. Many of the programs were small and took place in one health facility. All five articles which reported on programs in over 100 health facilities were about programs in rural settings in the Indian states of Bihar and Karnataka. The size and location of these programs raise interesting considerations for the application of lessons from the body of literature on nurse mentoring. Most of the published articles are about programs in a few facilities in high-income countries, but the programs in the largest number of facilities in the world are in India, a lower middle-income country. The level of engagement and management support required to mentor nurses across hundreds of facilities in a low- or middle-income country with fewer resources and limited infrastructure is higher than what is needed to run a mentoring program in one or two facilities in the United States.

Many articles did not specify the number of mentors and mentees in their programs or the frequency of contact and duration of mentoring, which made it difficult to summarize and discuss similarities and differences across programs. We could not categorize levels of nurse mentor and mentee experience or education because of the diversity among the program contexts. In many high-income countries, Bachelor of Science (BSc) education is entry. However, in Rwanda, where nine of the articles were from, many nurses have only a secondary school nursing degree, though this is changing [32]. In the Indian states where the nurse mentoring programs were implemented and eight of the articles were from, there are two levels of nurse midwives with either 18 months or 3 years of post-secondary education [40].

There were clear disagreements about how authors used the terms “precepting,” “mentoring,” and “clinical supervision.” Some specifically referred to preceptors as focused on clinical care and mentors on social support and personal growth [46–48]. Others referred jointly to “preceptorship-mentorship” roles [49]. Additional articles described the purposeful selection of mentors who were not in supervisory roles over mentees [42, 46, 50–52], while others noted that mentors were “nurse managers,” “ward managers,” or “charge nurses.” [47, 53–56]

Following the scoping review framework for this research, we did not make determinations about the quality of each included article or weight of overall evidence for specific policies or interventions [20]. If authors reported on their program as a nurse mentoring program or referenced mentoring as a key part of an intervention, we included the study (if it met the other previously discussed inclusion criteria). This differentiates our study from a recent scoping review of mentorship of health personnel in low- and middle-income countries, which focused on mentoring programs designed to improve quality of care in primary health care settings and excluded studies that did not meet their definition of mentoring [4].

Our inclusive approach meant the term “mentoring” was used in a variety of ways. The varieties of “nurse mentoring” made summarizing them challenging, but also meant that articles describing unique programs were included. In two articles about a nurse mentoring program to increase nurse retention, the program paid the mentors monetary incentives if mentees remained in the workforce at specific points [44] [45]. Another program formed an academic-clinical partnership between a nursing school and a hospital where many of its graduates worked to provide a nurse faculty mentor known to the mentees during their initial weeks on the job [57]. One program used retired nurses as mentors for younger nurses [58]. Our thorough review of the nurse mentoring literature and inclusion of a variety of author perspectives and program designs provides a well-rounded overview of current knowledge about nurse mentoring.

### Limitations

This study has several limitations. By choosing to include only articles about programs where nurses mentored other nurses, literature about interprofessional and multidisciplinary mentoring was excluded, which would have added alternate perspectives to the discussion, but made the dataset unwieldy. There were a number of articles about different facets of the same nurse mentoring programs or about iterations of the same program. We have attempted to manage this by describing those programs with multiple articles and their contributions clearly and reporting results on a per article rather than a per program basis. We attempted to reduce the risk of bias by defining clear criteria for selection of articles and thoroughly reviewing all articles at each level of the process. Any conflicts were discussed with co-authors to reach consensus. The total number of articles reviewed, particularly in the first screening stage, meant that decisions were made quickly based on the inclusion and exclusion criteria, which could have meant that some articles were overlooked.

## Conclusions

This scoping review provides an overview of the current literature on in-service nurse mentoring. Despite the limitations of existing literature, our review identified some informative themes and trends. It was striking how many articles did not specify key features of programs, such as mentor to mentee ratios, frequency of contact, and duration of mentoring. To understand which models of nurse mentoring work best in different settings, practitioners and researchers must conduct and publish additional research targeting these gaps to make models of nurse mentoring more transparent and translatable. The research about programs in rural settings indicates that mentoring may be a particularly useful intervention to strengthen clinical care, where nurses are geographically dispersed and may have fewer opportunities for formal or informal education or training. While many of the articles were recent and mostly from high-income countries, the nurse mentoring programs in the largest number of facilities are not in those countries and are steadily contributing more to the literature. Research on these large programs can provide information to strengthen implementation of large or complex nurse mentoring programs in other settings.

Nurse mentoring programs have the potential to strengthen the nursing workforce in a sustainable manner—from within the profession itself. By establishing what is known about in-service nurse mentoring and calling attention to remaining gaps, we hope this review will lead to new and groundbreaking research on workforce strengthening strategies that place nurses and midwives at the heart of responsive, people-centered health systems.

## Data Availability

The file generated by the data charting process and analyzed for this review are available from the corresponding author on reasonable request.

## Abbreviations

AMANAT: Emergency Obstetric and Neonatal Readiness (in Hindi) program
BSc: Bachelor of Science
HIV: Human immunodeficiency virus
MESH: Mentoring and Enhanced Supervision at Health Centers program
PRISMA: Preferred Reporting Items for Systematic Reviews and Meta-Analyses
